# DNA hypermethylation preceded by H3K27 trimethylation is linked to downregulation of gene expression in disuse muscle atrophy in male mice

**DOI:** 10.14814/phy2.70317

**Published:** 2025-04-13

**Authors:** Junya Shimizu, Fuminori Kawano

**Affiliations:** ^1^ Graduate School of Health Science Matsumoto University Nagano Japan

**Keywords:** disuse muscle atrophy, DNA methylation, epigenetic alterations, gene expression, H3K27me3

## Abstract

Disuse muscle atrophy can result in downregulated gene expression vital to muscle integrity, yet the mechanisms driving this downregulation remain unclear. Epigenetic alterations regulate transcriptional potential, with repressive changes suppressing gene expression. This study explored epigenetic mechanisms of gene downregulation during disuse muscle atrophy. Male C57BL/6J mice underwent hindlimb suspension for 3 or 7 days. The vastus intermedius (VI) muscle was analyzed, showing unchanged mass on day 3, but on day 7, decreased mass and reduced fiber size were assessed via immunohistochemistry. Corresponding to this atrophy timing, qPCR analysis revealed nine downregulated genes on day 7, which were selected for epigenetic analysis; collectively, they showed no downregulation on day 3. Among the nine genes, methylated DNA immunoprecipitation revealed significantly elevated DNA methylation (hypermethylation) in the upstream regions of transcription start sites (TSS) on day 7, which overall negatively correlated with gene expression. Histone marks (H3K27me3, H3K4me3, H3.3, and total H3) were also assessed using chromatin immunoprecipitation, revealing that the repressive histone mark H3K27me3 increased in the regions on day 3 but decreased on day 7. These findings suggest that DNA hypermethylation in the upstream regions preceded by H3K27me3 enrichment contributes to the downregulation of gene expression during disuse muscle atrophy.

## 1 INTRODUCTION

Disuse muscle atrophy, characterized by the loss of muscle mass and strength, often occurs during extended step reduction, limb immobilization, and bed rest, significantly impairing mobility and independence (Arentson‐Lantz et al., 2020; Holloway et al., 2019; McGlory et al., 2019). It is primarily caused by a reduced mechanical load on skeletal muscle, where muscle protein breakdown exceeds synthesis (Nunes et al., 2022). A central driver of this process is the activation of the FOXO transcription factor family, which translocates to the nucleus during reduced muscle activity and upregulates the expression of atrophy‐related genes, such as *Trim63* (Senf et al., 2010). Downregulation of the expression of certain protective genes also contributes to this process. For instance, Senf et al. found that, although heat shock protein 70 (*Hsp70*) expression declines in disuse atrophy, *Hsp70* overexpression counteracts this decline by inhibiting *Foxo3a* transcriptional activity and atrogene upregulation, thereby preventing further muscle degradation (Senf et al., 2008). Similarly, Cannavino et al. established that the expression of peroxisome proliferator‐activated receptor gamma coactivator 1‐alpha (*Pgc‐1α*), a mitochondrial biogenesis regulator, is downregulated during disuse and that its overexpression mitigates muscle loss by reducing autophagy, proteasome activity, and atrogene expression (Cannavino et al., 2014). Spradlin et al. further demonstrated that insulin‐like growth factor‐1 (*Igf1*) expression is downregulated during disuse and that muscle‐specific Igf1 deletion intensifies atrophy and weakness in mice (Spradlin et al., 2021). Despite these findings, the mechanisms underlying gene expression downregulation during disuse muscle atrophy remain poorly understood.

Epigenetic alterations such as DNA methylation and histone modifications play crucial roles in regulating gene expression without altering the DNA sequence. Repressive epigenetic marks, including DNA hypermethylation and trimethylation of lysine 27 on histone H3 (H3K27me3), downregulate gene expression by preventing transcription factor binding and compacting chromatin into a heterochromatin state (Buitrago et al., 2021; Cai et al., 2021). In skeletal muscle, these and other epigenetic modifications change dynamically with acute exercise, with some becoming stabilized through training, regulating gene expression and facilitating long‐term muscle adaptation (Shimizu & Kawano, 2022; Turner et al., 2019; Voisin et al., 2024). Skeletal muscle also undergoes epigenetic alterations in response to disuse, which coincide with the onset of atrophy. Fisher et al. found that the upregulation of atrogene expression is associated with decreased DNA methylation, a process that is reversible during recovery (Fisher et al., 2017). Tomiga et al. also reported that increased DNA methylation within the promoter region of a single gene is linked to its downregulation during disuse‐induced muscle atrophy (Tomiga et al., 2019). Although these findings suggest that shifting DNA methylation patterns contribute to gene expression changes during disuse muscle atrophy, few studies have examined epigenetic modifications in clusters of upregulated or downregulated genes in skeletal muscle. Moreover, the relationship between DNA methylation and histone modifications in downregulating gene expression during disuse muscle atrophy has not been well characterized. We hypothesize that DNA methylation is strongly associated with the downregulation of gene expression during disuse muscle atrophy and that repressive histone modifications are related to this process. To examine their relationship with the onset of muscle atrophy, the present study compares the epigenetic profiles of skeletal muscle between pre‐atrophic and atrophic conditions to assess their association with downregulation of gene expression during muscle atrophy.

## 2 MATERIALS AND METHODS

### 2.1 Ethical approval and animal care

All experimental procedures were conducted in accordance with the *Guide for the Care and Use of Laboratory Animals* of Matsumoto University (Nagano, Japan) and were approved by the Animal Use Committee of Matsumoto University (Approval ID: 2023–3). Male C57BL/6J mice aged 7 weeks were obtained from CLEA Japan (Tokyo, Japan). The mice were acclimated to the experimental environment for 1 week before the start of the experiments. To ensure consistency with established protocols and facilitate meaningful comparisons with previous skeletal muscle epigenetics studies on physical activity, 8‐week‐old mice were selected for this study (Ohsawa & Kawano, 2021; Shimizu et al., 2024; Shimizu & Kawano, 2022). Throughout the study, the animals were housed under controlled conditions, maintained at a temperature of 23°C, humidity of 40%–60%, and a 12‐h light–dark cycle. They were provided a commercial solid diet (CE‐2, Nihon CLEA) and water ad libitum. All experimental procedures adhered to the ARRIVE guidelines 2.0 (https://arriveguidelines.org/arrive‐guidelines).

### 2.2 Experimental design and muscle sampling

Eight‐week‐old mice were randomly assigned to either the control (*n* = 12) or unloaded (*n* = 12) groups. The unloaded group underwent hindlimb suspension (HLS) using the tail suspension method for either 3 (*n* = 6) or 7 days (*n* = 6), following the protocol described by Ohira et al. (Ohira et al., 2001). Briefly, mice were housed individually in cages measuring 22.5 × 33.8 cm with a height of 14.0 cm. Hindlimb suspension was achieved by securing a narrow piece of tape to the lower third of the tail using a cushion sponge placed underneath to relieve pressure and prevent injury. A second piece of tape was attached to the first piece and connected to a string that was tied to a horizontal bar at the top of the cage. The string was adjusted to elevate the hind limbs and prevent contact with the cage floor or walls while allowing the mice to move freely using their forelimbs. The control group was also individually housed in cages measuring 17.0 × 25.5 × 15.0 cm without hindlimb suspension. Both groups had ad libitum access to a commercial solid diet (CE‐2; Nihon CLEA) and water. After either 3 or 7 days of treatment, gastrocnemius and vastus intermedius muscle samples were collected from both control and unloaded groups. The gastrocnemius has been reported to undergo significant atrophy in response to hindlimb suspension in mice (Theilen et al., 2018), while hindlimb suspension has also been shown to induce atrophy of the vastus intermedius in rat models, accompanied by increased expression of type IIx and IIb myosin heavy chain isoforms (Adams et al., 1994). The mice were euthanized through exposure to gradually increasing concentrations of carbon dioxide gas in an inhalation chamber. Following euthanasia, the muscle tissues were carefully dissected, cleaned of excess fat and connective tissue, frozen in liquid nitrogen, and stored at −80°C for subsequent analysis.

### 2.3 Immunohistochemistry

Cross‐sectional immunohistochemical analyses were performed as described by Shimizu et al. (Shimizu & Kawano, 2022). Briefly, 10 μm‐thick cross‐sections were obtained from the mid‐belly of the vastus intermedius using a cryostat (Leica Microsystems, Wetzlar, Germany) set to −20°C. The vastus intermedius was specifically analyzed because its weight was significantly affected by HLS, unlike the gastrocnemius muscle. Sections were fixed in 4% paraformaldehyde for 5 min and subsequently blocked with 10% goat or donkey serum in PBS containing 0.1% Triton X‐100 (TPBS) for 20 min. After blocking, the sections were incubated overnight at 4°C with the following primary antibodies: anti‐dystrophin (ab129996; Abcam, Cambridge, UK), anti‐type IIb MyHC (BF‐F3; Developmental Studies Hybridoma Bank), and anti‐type IIa MyHC (SC‐71; Developmental Studies Hybridoma Bank), each diluted 1:100 in TPBS containing 1% bovine serum albumin. The next day, Alexa Fluor 350‐conjugated goat anti‐mouse IgG2b cross‐adsorbed secondary antibody (A‐21140; Thermo Fisher Scientific, Waltham, MA, USA) was used to detect anti‐dystrophin, Alexa Fluor 546‐conjugated goat anti‐mouse IgM (heavy chain) cross‐adsorbed secondary antibody (A‐21045; Thermo Fisher Scientific) was used to detect anti‐type IIb MyHC, and Alexa Fluor 488‐conjugated goat anti‐mouse IgG1 cross‐adsorbed secondary antibody (A‐21121; Thermo Fisher Scientific) was used to detect anti‐type IIa MyHC. Each secondary antibody was diluted 1:500 in TPBS containing 1% bovine serum albumin and incubated for 1 h to visualize primary antibody binding. The stained sections were mounted using an antifade mounting medium (H‐1000; Vector Laboratories) for microscopy. Whole‐section images were acquired using an all‐in‐one fluorescence microscope (BZ‐X710; Keyence, Osaka, Japan) with consistent exposure times to ensure comparable staining across sections. The whole sections were imaged using a tile‐scanning mode to cover the entire area with high resolution. Muscle fiber phenotypes were determined based on the fluorescence intensity of each MyHC isoform, with fibers classified as either type IIb or IIa. Fibers not labeled with these antibodies were classified as type IIx, as previously reported (Sandona et al., 2012). Fiber type and size were analyzed using a semiautomated approach with BZ‐X Analyzer software (Keyence), where thresholds for fluorescence intensity were manually set based on the generated distribution maps. The area enclosed by dystrophin (muscle fiber size) was also analyzed in all images. For the analysis of fiber phenotype and size, approximately 2000 fibers per muscle section were examined.

### 2.4 RNA extraction

A piece of frozen muscle sample (10–20 mg) was homogenized in 1 mL of ISOGEN (311–02501, NIPPON GENE, Toyama, Japan) for total RNA extraction, following the manufacturer's instructions. RNA was isolated manually using this reagent, and its concentration and purity were determined spectrophotometrically using a NanoDrop spectrophotometer (Thermo Fisher Scientific). The A260/280 ratios were between 1.8 and 2.0, and the A260/230 ratios exceeded 2.0, indicating high‐quality RNA. Total RNA (800 ng) was then used to synthesize cDNA using SuperScript VILO Master Mix (11,755,050, Thermo Fisher Scientific). The mixture was incubated at 42°C for 60 min, followed by enzyme inactivation at 85°C for 5 min. The synthesized cDNA was subsequently diluted to a 1/100 concentration with ultrapure water and stored at −20°C until analysis.

### 2.5 Chromatin extraction

Chromatin was extracted as described by Kawano et al. (Kawano et al., 2015). Briefly, frozen muscle samples (40 mg) were homogenized in chilled PBS and centrifuged at 12,000 ***g*** to pellet the chromatin. The pellet was fixed in 1% paraformaldehyde, sonicated using a Sonifier 250 (Branson, Danbury, CT, USA), and subjected to gel filtration using a commercial kit (7,326,300, Bio‐Rad, Hercules, CA, USA) to remove non‐nucleosomal small DNA and free histones. The resulting chromatin‐rich extract was adjusted to a fixed concentration containing 500 ng of DNA per reaction (50 μL/reaction) and stored at −80°C for further analysis.

### 2.6 Chromatin immunoprecipitation (ChIP)

The chromatin samples were incubated with the following specific antibodies: anti‐H3K27me3 (9733, Cell Signaling Technology, Danvers, MA, USA, 1:50), anti‐H3K4me3 (9751, Cell Signaling Technology, 1:50), anti‐H3.3 (ab176840, Abcam, 1:50), and anti‐total H3 (4620, Cell Signaling Technology, 1:50) for 1 h at 4°C. The samples were then incubated with Dynabeads™ Protein A (10002D, Thermo Fisher Scientific) for 30 min at 4°C. The protein complexes were then washed and digested with proteinase K (9034, Takara Bio, Shiga, Japan) for 1 h at 65°C. DNA was extracted from the samples and resuspended in tris‐EDTA buffer. To ensure accuracy, ChIP reactions were repeated twice, and the DNA yields were combined within each group. The amount of input DNA used in each ChIP reaction was determined without immunoprecipitation.

### 2.7 Methylated DNA immunoprecipitation (MeDIP)

Chromatin‐rich extracts from each experimental group were pooled to obtain 500 ng of DNA per reaction (50 μL/reaction). DNA was extracted and incubated with an anti‐5‐methylcytosine antibody (5mC) (ab214727, Abcam, 1:50) following the same procedure used for ChIP. To validate the MeDIP assay, we examined negative and positive control samples. For the negative control, chromatin samples were incubated with normal rabbit IgG (2729, Cell Signaling Technology; 1:50 dilution). For the positive control, DNA fragments from the chromatin samples were incubated with CpG methyltransferase (M.SssI, M0226S, New England Biolabs) before the MeDIP reaction. As with the ChIP reactions, the DNA yields from repeated MeDIP reactions were combined within each group.

### 2.8 Quantitative PCR (qPCR)

qPCR was performed using the StepOne Real‐Time PCR System (Thermo Fisher Scientific) with THUNDERBIRD SYBR qPCR Mix (QPX201, TOYOBO, Osaka, Japan) following the manufacturer's instructions. Each reaction was carried out in a total volume of 20 μL, consisting of 10 μL of THUNDERBIRD SYBR qPCR Mix, 0.4 μM of each primer, and 2 μL of cDNA template synthesized from 800 ng of total RNA. The PCR cycle settings were as follows: initial denaturation at 95°C for 30 s, followed by 40 cycles of denaturation at 95°C for 30 s, and annealing/extension at 60°C for 30 s. The primer pairs used for gene expression analysis and the ChIP/MeDIP assays are listed in Tables 1 and 2, respectively. Initially, 21 genes were selected based on previous studies reporting reduced expression in skeletal muscles under unloading conditions, such as HLS, bed rest, and immobilization (Table 1) (Cannavino et al., 2014; Chen et al., 2022; Kaneguchi et al., 2014; Kotani et al., 2022; Rullman et al., 2018; Segales et al., 2020; Senf et al., 2008; Sharlo et al., 2021; Spradlin et al., 2021; Theilen et al., 2018; Vitadello et al., 2014, 2020; Xu et al., 2022; Yasuhara et al., 2011). Of these, nine genes that were significantly downregulated on day 7 post‐HLS were selected for further epigenetic analysis (Table 2). Similarly, 21 genes were extracted from muscle atrophy‐related genes identified as FOXO‐dependent in a previous study (Table 1) (Oyabu et al., 2022). From these, nine genes exhibiting significant upregulation on day 7 post‐HLS were selected for subsequent epigenetic analysis (Table 2). Primer pairs for ChIP‐qPCR were designed 500 bp upstream and downstream of the transcription start site (TSS) for the nine target genes. qPCR results were quantified by normalizing the cycle threshold (Ct) values of target gene amplification to *Gapdh* mRNA as the internal control for gene expression assays, or by normalizing the Ct values of the respective input for ChIP‐qPCR (% input). The coefficient of variation (%CV) for Gapdh Ct values was <2%, indicating stable expression and consistent input normalization, with no significant effect of HLS. The data were further normalized by averaging the mean values for each position (upstream and downstream of the TSS, 500 bp each) across each locus. The downstream region of Vegfa was excluded from the MeDIP analysis, and both *Pgc‐1α* and *Vegfa* downstream regions were excluded from the ChIP analysis because of amplification failure.

**TABLE 1:** Candidate factors and primer sequences for gene expression analysis.

Gene symbol	Forward primer	Reverse primer
*Atg14*	cagaaagaagtactaaaagctatggaagg	gtttgcttcagctgttcaatcct
*Atrogin‐1*	agtgaggaccggctactgtg	gatcaaacgcttgcgaatct
*Cathepsin L*	tctcacgctcaaggcaatca	aagcaaaatccatcaggcctc
*Ccnd1*	agaagtgcgaagaggaggtc	ttctcggcagtcaagggaat
*Colq*	gtgtgacccctaccagaaca	cccaaaccagagaaaccagc
*Csrp3*	tcactgacaaggatggggag	gctacaaaggaggctgttgg
*EIF4EBP1*	ggcggcacgctcttca	tccgacactccatcagaaatttc
*Fbxl3*	gcgacatcttacttgaaagccac	ttgctgctgtccaccttgaa
*Fbxo9*	tctaccgtagggcgatgca	ctcttcgatgtagccgctcc
*FIP200*	cacagtatctggcggatcaaca	tcagcgtggttccggtgt
*Gabarapl1*	cactgttggccagttctacttcttaa	cactggtgggagggatggt
*Gadd45a*	cgtagaccccgataacgtggta	cggatgagggtgaaatggat
*Gapdh*	caaggacactgagcaagaga	gcccctcctgttattatggg
*Glul*	gcgaagactttggggtgata	actggtgcctcttgctcagt
*HSF2*	aatttggaatccctgcaccg	ctcacaaagctcgccatgtt
*Hsp90b1*	acacctcagaagacgcagaa	ttacagtgcaggggagaagg
*Hspa1a*	tgctgatccaggtgtacgag	cgttggtgatggtgatcttg
*Hspa1b*	tgctgatccaggtgtacgag	cgttggtgatggtgatcttg
*Hspa8*	tctaagggacctgcagttgg	ttgcaacctgattcttggcc
*Hspd1*	tagctgttacaatggggcca	ggcaacgtcctgaacaagtt
*Igf1*	gtcacacaaactcaccaccc	ttctgatgttgcaccctcct
*Igfbp4*	ggaaaggaatggggtgagga	aatatggggacggaggcaaa
*Itgb1bp2*	agtcttggagctgttgtggt	aagcggaatctggccataga
*Klhl21*	gacgggcgggtctgatg	tctcgggccttgagcatg
*Klhl22*	gcaggccgtgactaccaca	gcatatacctccttcttgagtggg
*Klhl40*	ccatcttcaccatctgcgtg	aacactgcctcctccttctc
*LC3b*	gcttgcagctcaatgctaac	cctgcgaggcataaaccatgt
*Lonp1*	ccaacatccttaagcggctc	tccagccccaattccttctt
*Myc*	tcagacacggaggaaaacga	cgtctgcttgaatggacagg
*Nmrk2*	agatgttctgactggacgca	agggaaactgaggcacatca
*Nos1*	gtccgattcaacagcgtctc	gctcatctccctccctcatc
*p62*	tctggggtagtgggtgtcag	agaatgtgggggagagtgtg
*Pdk4*	agctggtgaagagctggtatatcc	tctggtcttctgggctcttctc
*Pgc‐1a*	gcttgactggcgtcattcg	acagagtcttggctgcacatgt
*Psma1*	cattggaatcgttggtaaagac	gttcatcggctttttctgc
*Psmc2*	tggaggaaaaacccgacgt	tctctggatggagcagaggg
*Psmd11*	gagttccagagagcccagtc	aacccagttcaaggatgctc
*Psmd3*	gttggcttcaaacagacggtg	cgctttagggagggctgg
*Sesn1*	accactctcttgcctccttc	ctcggcattcctgtaactgc
*Tfam*	cttcaaccaccacaccactg	tcctccatgcgttcttctgt
*Trim63*	cctgcagagtgaccaagga	ggcgtagagggtgtcaaact
*Ulk1*	tgaagcaggtggtacgcaga	gccgttgtttgtccagaaaga
*Vegfa*	gctgtaacgatgaagccctg	cgctccaggatttaaaccgg

**TABLE 2:** Primers for ChIP‐qPCR and MeDIP‐qPCR analyses.

Gene symbol	Position from TSS	Forward primer	Reverse primer
*Hsp90b1*	−500 bp +500 bp	ggacaagcccaatcgcaag atgagggtcctgtgggtgtt	taagctcctcacacctccag ggaacgagatggtccctgaa
*Cathepsin L*	−500 bp +500 bp	gcaaatctcagcaaccacat ccccctctctaaccctca	agtcattggaacaggggtaaa cctcccgaaaacacacaaag
*EIF4EBP1*	−500 bp +500 bp	atcagcccaccctttcttct ctcggttgctcgccttct	gcttctgtcctctccagtgc atcttgtcccaccccaaact
*Fbxo9*	−500 bp +500 bp	cacccctccccttttacct tgattgttgagttttcgctctc	gacgctacattcacccgact ccgcctgtttgatgctga
*Hspa8*	−500 bp +500 bp	gtccccatctgcacccatag aagggttgcagactttctcca	ttggcaccatacagttgtcct ggcagtgttggcagttacctt
*Hspd1*	−500 bp +500 bp	ccttgcttgcaccaacttga agtccttcgccagatgagac	ctgtcctctgctaaccacca tggccccattgtaacagcta
*Igf1*	−500 bp +500 bp	gagagaaggcgaatgttccc ggggaaaatcagcagccttc	aaatgaattggtgggcaggg ccttttacagctcgcacaga
*Igfbp4*	−500 bp +500 bp	aaggtttctcttctcccgtgg gtgacactgccaagacaagg	ccccaagaggtctgagtgta tgcctctggaagctctgtag
*Lonp1*	−500 bp +500 bp	tcctgctttctccgtcatgt agcgacgagacctccgag	ataaggtgagtgagtgggcg gataaagcgcgggaacacag
*p62*	−500 bp +500 bp	ctggatgaaggggaagtctg gctaccgtcaccctcgtg	aggctgggaaggtagaaacc aagcctcctgtccttctcct
*Pdk4*	−500 bp +500 bp	tcctagcgacctgggatcta gagctgttctcccgctacag	ctagaaaggcctggcacagt cagtcgtcctagccctgaac
*Pgc‐1a*	−500 bp +500 bp	agcgttacttcactgaggca catgtgcagccaagactctg	tgaactgaaggcacctgtct ccgctgagccactttctatg
*Psma1*	−500 bp +500 bp	acacacacacacacacacacac gtgtgttgagccccgagt	gcccagagtttccttgcttt gccgcccaggttactgat
*Psmc2*	−500 bp +500 bp	cacactccctaacagaaatgc gaggaggagaaggacgacaa	cgctccctcacaactcca ggcagacagacagacagacg
*Psmd11*	−500 bp +500 bp	ggtgctattcctgccacata tcctccactccatcggtaaa	cgggagttgtagtctctctgc cctctcaacaacacactctcc
*Tfam*	−500 bp +500 bp	gcggactcatgaaacatgca caggatgccacgacttgttt	tggtctaaggtgggtgttgc taaaagaggttgcagggctg
*Trim63*	−500 bp +500 bp	atccaggaggggaacagatt gacagtcgcatttcaaagca	gggggtcgtaaacaagaaca gcctagcactgacctggaag
*Vegfa*	−500 bp +500 bp	gcagactattcagcggactc gacggacagacagacagaca	gaaccaacctcctcaaaccg tactacggagcgagaagagc

### 2.9 Statistical analysis

Statistical analyses were performed using BellCurve for Excel (Social Survey Research Information Co., Ltd., Tokyo, Japan). Comparisons between control and HLS groups were performed separately at each time point (Day 3 and Day 7). No statistical comparisons were made across time points, as each dataset was obtained and processed independently. For all comparisons between two groups, we first assessed the equality of variances using an F‐test. If equal variances were assumed (*p* > 0.05), Student's *t*‐test was applied; otherwise, the Mann–Whitney *U* test was used. Spearman's rank‐order correlation was also used to evaluate the relationships between gene expression, DNA methylation, and histone modifications, as these variables do not necessarily exhibit linear relationships. Statistical significance was set at *p* < 0.05.

To assess representative epigenetic changes, gene expression and ChIP/MeDIP analyses were conducted on groups of genes rather than on individual genes. Given the complexity of epigenetic regulation, analyzing individual genes independently can be influenced by biological variability among individuals and inherent differences in gene‐specific regulation, which may reduce the consistency of observed trends. Instead, pooling data from functionally related gene groups allows for a more consistent and interpretable assessment of epigenetic modifications that contribute to muscle atrophy. A similar approach has been employed in previous epigenetic studies, including our own, to reduce variability and detect broader regulatory patterns that may not be evident when analyzing single genes in isolation (Masuzawa et al., 2024; Ohsawa & Kawano, 2021; Shimizu et al., 2024).

## 3 RESULTS

### 3.1 Muscle weights and morphology

**FIGURE 1:**
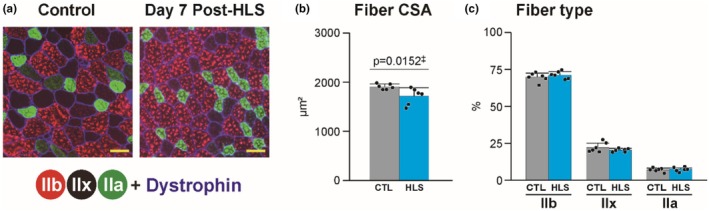
Effects of HLS on fiber size and types in mouse VI muscle on day 7. (a) Muscle sections from the vastus intermedius stained for type IIb (red), IIa (green) myosin heavy chain isoforms, and dystrophin (blue). Type IIx fibers are not stained by any myosin antibodies. Scale bars = 50 μm. (b) Cross‐sectional area (CSA) of muscle fibers. (c) Percentage distribution of fiber types. All values are normalized to those of the control group. Data are presented as the mean ± SD, with individual data points for biological replicates. †Student's *t*‐test; ‡Mann–Whitney *U* test.

To examine temporal differences in disuse muscle atrophy and distinguish between pre‐atrophic and atrophic conditions, we analyzed both the VI and gastrocnemius muscles. HLS resulted in a progressive reduction in body mass, with significant declines observed on day 3 (−7.5%, *p* = 0.0026) and day 7 (−16.1%, *p* = 0.0036) (Table 3). The relative weight of the gastrocnemius muscle significantly decreased on day 3 (−4.5%, *p* = 0.0152), with a further reduction observed on day 7 (−12.2%, *p* = 0.0004) (Table 3). For the VI muscle, a significant reduction in absolute weight was observed on both day 3 (−10.9%, *p* = 0.0007) and day 7 (−25.4%, *p* < 0.0001) (Table 3). However, while a significant reduction in relative weight was detected on day 7 (−13.0%, *p* = 0.0006), no such reduction was observed on day 3 (Table 3). These findings demonstrate that the observed changes in muscle weight distinguish between pre‐atrophic and atrophic conditions and reflect temporal differences associated with disuse muscle atrophy. Therefore, we focused all subsequent analyses on the VI muscle. Because significant atrophy was first observed on day 7 post‐HLS, immunohistochemical analysis was performed exclusively on the VI muscle at this time. The analysis on day 7 revealed a significant 10% reduction in the cross‐sectional area (CSA) of myofibers (*p* = 0.0152), without alterations in fiber type composition (Figure 1a–c).

**TABLE 3:** Body mass and hindlimb muscle weight.

	3 days			7 days		
	CON	HLS	Δ	CON	HLS	Δ
Body weight, g	22.4 ± 0.7	20.7 ± 1.1	−7.5%**^a^	22.1 ± 0.8	18.5 ± 2.5	−16.1%**^a^
Gastrocnemius						
Absolute weight, mg	127.5 ± 3.6	112.6 ± 10.1	−8.9%**^a^	113.3 ± 3.0	83.2 ± 15.0	−29.4%**^a^
%BW × 10^3^	5.69 ± 0.07	5.43 ± 0.23	−4.5%*^b^	5.14 ± 0.11	4.52 ± 0.33	−12.2%**^a^
Vastus intermedius						
Absolute weight, mg	76.9 ± 2.9	69.2 ± 4.3	−10.9%**^a^	76.1 ± 5.7	55.3 ± 10.7	−25.4%**^a^
%BW × 10^3^	3.43 ± 0.10	3.34 ± 0.06	−2.5%	3.45 ± 0.16	3.00 ± 0.22	−13.0%**^a^

**p < 0.01 and *p < 0.05; CON vs. HLS.

*Note*: Mean ± SD.

^a^ Student's *t*‐test;

^b^ Mann–Whitney *U* test.

### 3.2 Gene expression

**FIGURE 2:**
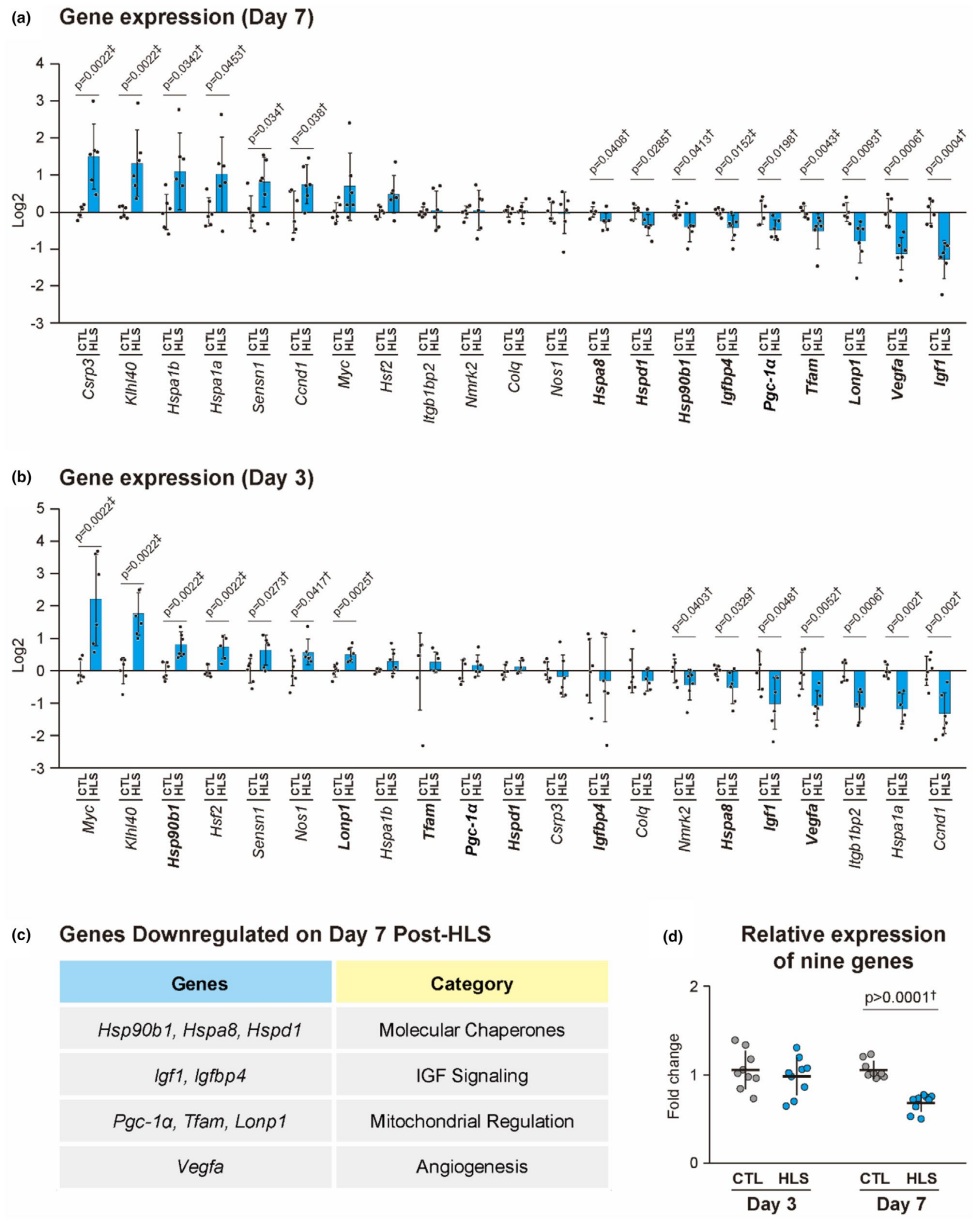
Effects of HLS on gene expression in mouse VI muscle. (a) Gene expression of 21 candidate genes on day 7 post‐HLS. (b) Gene expression of 21 candidate genes on day 3 post‐HLS. Genes shown in bold represent those that were significantly downregulated on Day 7 post‐HLS. (c) Categorization of downregulated genes based on their biological functions. (d) Mean expression levels of nine genes significantly downregulated on day 7 post‐HLS. Values are normalized to those of the control group. Panels (a) and (b) display log2 fold changes relative to the control and panel (d) shows fold changes. Data are presented as the mean ± SD, with individual data points for biological replicates (a, b) and average values for each gene (D). †Student's *t*‐test; ‡Mann–Whitney *U* test.

To identify epigenetic targets involved in the downregulation observed in disuse atrophy, we analyzed the expression levels of 21 candidate genes in VI muscle samples on day 7 post‐HLS. These 21 genes were identified based on prior studies demonstrating their altered expression under unloading conditions and their roles in pathways critical for muscle maintenance, including chaperone function, IGF signaling, mitochondrial integrity, and angiogenesis (Cannavino et al., 2014; Chen et al., 2022; Kaneguchi et al., 2014; Kotani et al., 2022; Rullman et al., 2018; Segales et al., 2020; Senf et al., 2008; Sharlo et al., 2021; Spradlin et al., 2021; Theilen et al., 2018; Vitadello et al., 2014, 2020; Xu et al., 2022; Yasuhara et al., 2011). Among these, nine genes (*Hsp90b1*, *Hspa8*, *Hspd1*, *Igf1*, *Igfbp4*, *Lonp1*, *Pgc‐1α*, *Tfam*, and *Vegfa*) showed significant downregulation on day 7 post‐HLS and were selected for further analysis (Figure 2a,c). Conversely, six genes (*Csrp3*, *Klhl40*, *Hspa1b*, *Hspa1a*, *Sensn1*, and *Ccnd1*) were significantly upregulated at the same time point. On day 3 post‐HLS, three of the downregulated genes (*Igf1*, *Vegfa*, and *Hspa8*) already showed significant reductions, whereas two (*Hsp90b1* and *Lonp1*) exhibited significant increases in expression (Figure 2b). Analysis of the aggregate expression levels across the nine downregulated genes showed no significant changes on day 3, whereas their expression levels were markedly reduced on day 7 (−33%, *p* = 0.0001) (Figure 2d). These nine genes with significant downregulation on day 7 post‐HLS were further examined for DNA methylation and histone modifications.

### 3.3 DNA methylation

**FIGURE 3:**
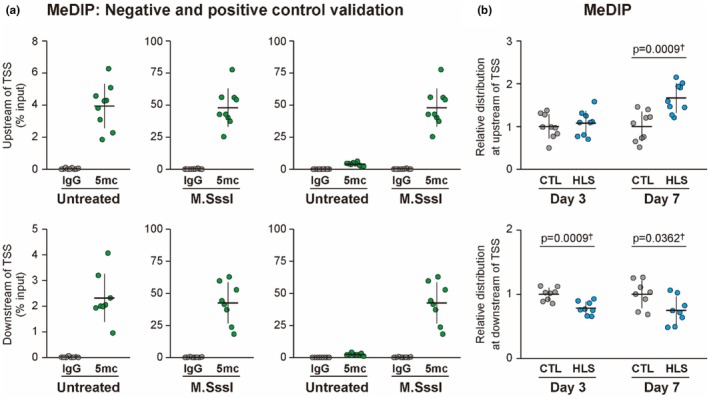
DNA methylation levels around the TSS of nine genes downregulated on day 7 post‐HLS in mouse VI muscle. (a) Validation of the MeDIP assay: The MeDIP assay was validated using negative and positive controls. Normal rabbit IgG served as the negative control, and CpG methyltransferase‐treated DNA was used as the positive control. DNA methylation levels were analyzed in the regions upstream (−500 bp, upper row) and downstream (+500 bp, lower row) of the TSS of downregulated genes. (b) DNA methylation levels around the TSS: DNA methylation levels were measured in the upstream (−500 bp) and downstream (+500 bp) regions of the TSS for downregulated genes on days 3 and 7 post‐HLS. Each dot represents the DNA methylation level for an individual gene, derived from pooled samples in each experimental group. Values are normalized to the control group and presented as the mean ± SD. †Student's *t*‐test; ‡Mann–Whitney *U* test.

Control tests using SssI methylase and IgG confirmed the high specificity of the antibodies for MeDIP, which effectively precipitated the methylated DNA (Figure 3a). In the region upstream of the TSS of the genes with downregulated expression, no significant differences in DNA methylation were observed between the HLS and control groups on day 3 (Figure 3b). However, on day 7, DNA methylation in this region was significantly increased in the HLS group (+66%, *p* = 0.0009). In the downstream region of the TSS, DNA methylation significantly decreased in the HLS group on days 3 (−21%, *p* = 0.0009) and 7 (−25%, *p* = 0.0362) (Figure 3b).

### 3.4 Histone modification

**FIGURE 4:**
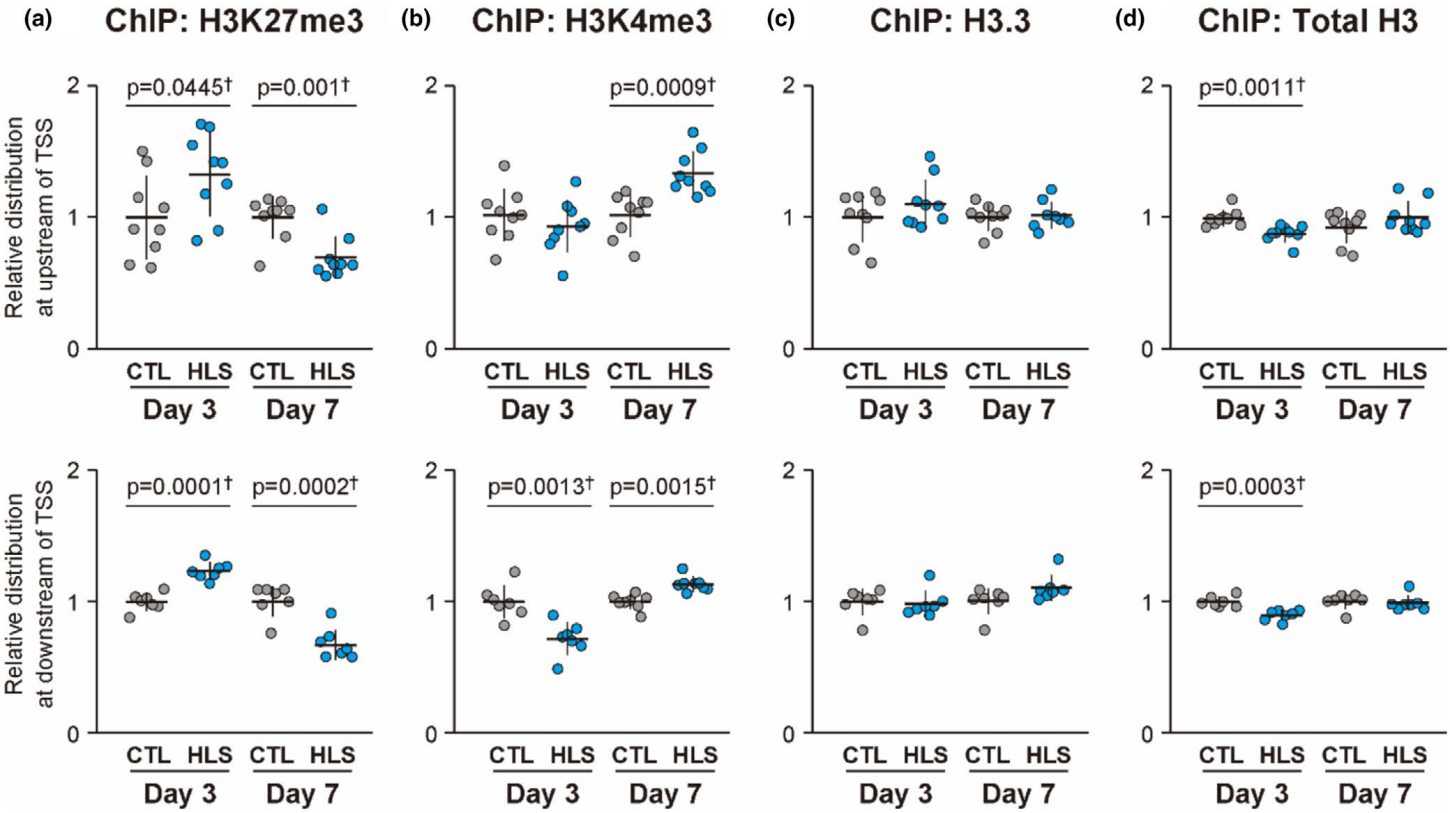
Histone mark levels around the TSS of nine genes downregulated on day 7 post‐HLS in mouse VI muscle. (a–d) Histone marks were analyzed around the TSS on days 3 and 7 post‐HLS. Antibodies targeting (a) H3K27me3, (b) H3K4me3, (c) H3.3, and (d) total H3 were used. Levels were measured upstream (−500 bp, upper row) and downstream (+500 bp, lower row) of the TSS. Each dot represents the histone mark level for an individual gene, calculated from experimental replicates in each group. Values are normalized to the control group and presented as the mean ± SD. †Student's *t*‐test; ‡Mann–Whitney *U* test.

In the HLS group, H3K27me3 levels increased significantly on day 3 (+33%, *p* = 0.0445) in the upstream region of the TSS and similarly increased in the downstream region (+24%, *p* = 0.0001). On day 7, H3K27me3 levels decreased in both regions (upstream: −31%, *p* = 0.001; downstream: −33%, *p* = 0.0002) (Figure 4a). H3K4me3 levels showed no significant changes on day 3 in the upstream region but decreased in the downstream region (−28%, *p* = 0.0013). On day 7, H3K4me3 levels increased significantly in both regions (upstream: +32%, *p* = 0.0009; downstream: +13%, *p* = 0.0015) (Figure 4b). H3.3 levels showed no significant changes in either the upstream or downstream regions on day 3 or day 7 (Figure 4c). Total H3 levels decreased significantly on day 3 in both regions (upstream: −12%, *p* = 0.0011; downstream: −10%, *p* = 0.0003), with no significant changes observed on day 7 in either region (Figure 4d).

### 3.5 Correlation analysis

**FIGURE 5:**
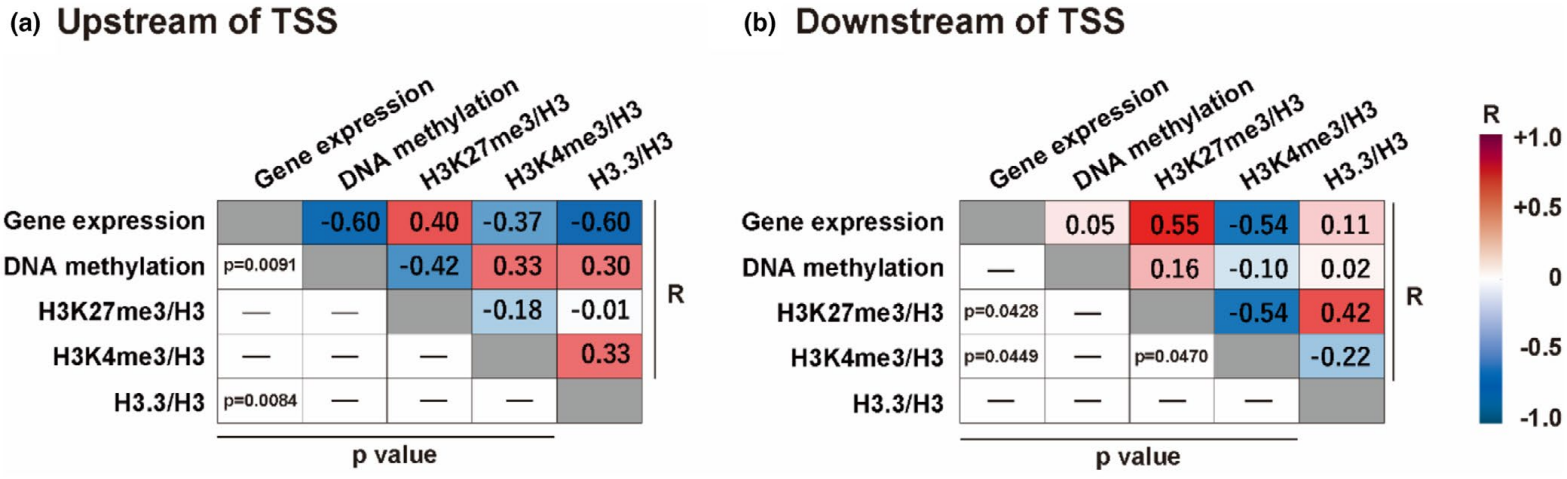
Correlation among gene expression, DNA methylation, and histone marks in mouse VI muscle. (a) Correlation analysis upstream (−500 bp, left) and (b) downstream (+500 bp, right) of the TSS. Spearman's rank‐order correlation was used to assess the relationships between gene expression, DNA methylation (Figure 2), and histone modifications (Figure 3, normalized to total H3 levels). The correlation strength and direction are indicated by the correlation coefficient (R).

Correlation analysis revealed a significant negative correlation between DNA methylation levels and gene expression in the upstream region of the TSS (*p* = 0.0092) (Figure 5a). Gene expression also showed a negative correlation with H3.3 levels in the upstream region (*p* = 0.0084). In the downstream region, gene expression was positively correlated with H3K27me3 levels (*p* = 0.0429) and negatively correlated with H3K4me3 levels (*p* = 0.0449) (Figure 5b). A significant negative correlation was observed between H3K4me3 and H3K27me3 levels in the downstream region (*p* = 0.047) (Figure 5b).

### 3.6 DNA methylation in upregulated genes

**FIGURE 6:**
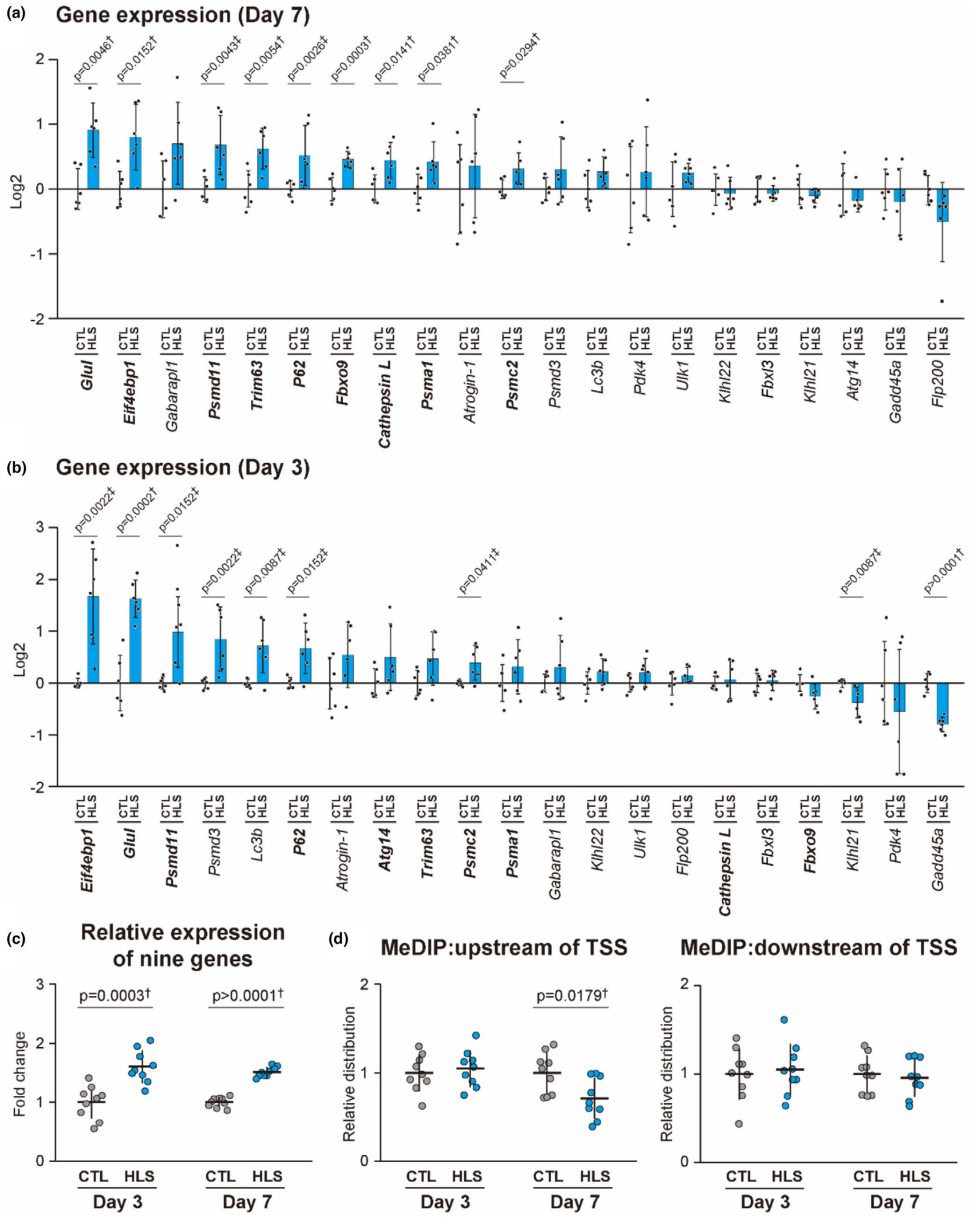
DNA methylation levels and gene expression of nine genes upregulated on day 7 post‐HLS in mouse VI muscle. (a) Gene expression of 21 candidate genes on day 7 post‐HLS. (b) Gene expression of 21 candidate genes on day 3 post‐HLS. Genes shown in bold represent those that were significantly upregulated on Day 7 post‐HLS. (c) Mean expression levels of nine genes significantly upregulated on day 7 post‐HLS. (d) DNA methylation levels in the upstream (−500 bp, left) and downstream (+500 bp, right) regions of the TSS of nine upregulated genes, measured by MeDIP assay on days 3 and 7 post‐HLS. Values are normalized to those of the control group. Panels (a) and (b) display log2 fold changes relative to the control, and panels (c) and (d) show fold changes. Data are presented as the mean ± SD. For panels (a) and (b), each dot represents the value from a biological replicate. In panel (c), each dot represents the mean expression level of an individual gene across biological replicates. In panel (d), each dot represents the DNA methylation level of an individual gene, derived from pooled chromatin samples within each experimental group. †Student's *t*‐test; ‡Mann–Whitney *U* test.

Given the significant negative correlation between gene expression and DNA methylation levels upstream of the TSS (Figure 5a), we investigated whether upregulated genes during disuse atrophy exhibit hypomethylation in this upstream region, in contrast to the hypermethylation observed in downregulated genes. To address this, we analyzed 21 muscle atrophy‐related genes regulated by FOXO signaling (Oyabu et al., 2022). These genes were selected based on their established roles in promoting protein degradation and suppressing protein synthesis that contribute to the progression of muscle atrophy (Oyabu et al., 2022). Among these, nine genes (*Cathepsin L*, *Eif4ebp1*, *Fbxo9*, *Glul*, *Psma1*, *Psmc2*, *Psmd11*, *p62*, and *Trim63*) were significantly upregulated on day 7 post‐HLS (Figure 6a) and were selected as target genes for MeDIP analysis. On day 3 post‐HLS, five of the target genes (*Eif4ebp1*, *Glul*, *Psmd11*, and *p62*) already exhibited a significant increase in expression (Figure 6b). Analysis of the aggregate expression levels across the nine upregulated genes revealed that their expression levels were significantly elevated on both day 3 (+60%, *p* = 0.0003) and 7 post‐HLS (+51%, *p* < 0.0001) (Figure 6c). DNA methylation levels in the upstream and downstream regions of the TSS showed no significant changes on day 3 (Figure 5d). On day 7, however, DNA methylation levels significantly decreased in the upstream region of the TSS (−29%, *p* = 0.0179) (Figure 6d).

## 4 DISCUSSION

### 4.1 Downregulation of genes linked to muscle atrophy

The present study demonstrated that HLS resulted in significant muscle atrophy in the vastus intermedius on day 7, accompanied by the downregulation of the expression of nine key genes involved in pathways essential for muscle maintenance (Figure 1a–c). Specifically, the expression of molecular chaperones, such as *Hsp90b1*, *Hspa8*, and *Hspd1*, was markedly decreased on day 7 post‐HLS (Figure 2a). These chaperones play critical roles in protein refolding and cellular homeostasis, and their downregulation compromises the ability of muscles to recover from cellular stress, leading to impaired protein repair and loss of structural integrity (Kruger et al., 2019; Marino Gammazza et al., 2018; Senf, 2013). Concurrently, we observed a significant reduction in the expression of genes involved in the IGF signaling pathway, including *Igf1* and *Igfbp4* (Figure 2a), which are key regulators of protein synthesis and inhibitors of protein degradation in the skeletal muscle (Maridas et al., 2017; Spradlin et al., 2021). Furthermore, the expression of genes essential for maintaining mitochondrial integrity, such as *Pgc‐1α*, *Tfam*, and *Lonp1*, was downregulated (Figure 2a). *Pgc‐1α* is a master regulator of mitochondrial biogenesis that inhibits muscle loss by suppressing FOXO activation and reducing oxidative stress (Kang et al., 2015; Sandri et al., 2006). *Tfam* is critical for protecting mitochondrial DNA from oxidative damage and promoting mitochondrial function (Theilen et al., 2017). *Lonp1* is a key protease responsible for maintaining mitochondrial quality control by eliminating damaged proteins, thereby preventing further loss of muscle mass and strength during periods of disuse (Xu et al., 2022). Additionally, we observed a reduction in the expression of *Vegfa*, a gene essential for promoting angiogenesis in the skeletal muscle (Figure 2a). *Vegfa* plays a pivotal role in increasing blood flow, enhancing nutrient delivery, reducing connective tissue accumulation, and maintaining muscle fiber content (Olfert et al., 2009). Collectively, the downregulation of the expression of these genes highlights disruptions in critical pathways, such as protein homeostasis, mitochondrial function, and angiogenesis, all of which are essential for muscle maintenance. This downregulation coincided with the progression of muscle atrophy on day 7, suggesting that these disrupted pathways may be involved in the development of disuse muscle atrophy.

The overall gene expression profile on day 3 exhibited variability, with no consistent downregulation pattern (Figure 2b,d). This is consistent with the absence of atrophy in the vastus intermedius muscle at this early stage (Table 3). Similarly, FOXO‐dependent atrophy genes, which are key regulators of muscle degradation, were significantly upregulated on day 7 post‐HLS, including *Trim63* (MuRF1), but remained unchanged on day 3 (Figure 6a,b). MuRF1, an E3 ubiquitin ligase, is a key mediator of disuse muscle atrophy, and its knockout attenuates hindlimb muscle loss in mice subjected to hindlimb suspension and sciatic nerve denervation, underscoring its critical role in muscle protein breakdown (Bodine et al., 2001; Labeit et al., 2010). Cannavino et al. further showed that HLS induced gastrocnemius muscle atrophy on day 3 in mice, coinciding with MuRF1 expression, which peaked on day 3 post‐HLS before returning to baseline on day 7 (Cannavino et al., 2015). These observations suggest a coordinated regulatory mechanism, in which the upregulation of FOXO‐dependent atrophy genes and the suppression of homeostasis‐maintaining genes together may be involved in the progression of muscle atrophy.

### 4.2 Epigenetic regulation of gene expression in disuse muscle atrophy

DNA hypermethylation was observed within the 500 bp upstream regions of the TSS in downregulated genes on day 7 post‐HLS, coinciding with reduced gene expression (Figure 3a). These upstream regions, which contain key regulatory elements such as transcription factor binding sites and core promoters, are critical for recruiting transcriptional machinery and initiating transcription (Lenhard et al., 2012). DNA methylation in these regions typically represses transcription by preventing transcription factor binding (Jones, 2012; Kaluscha et al., 2022). Consistent with this mechanism, an inverse correlation between gene expression and DNA methylation levels was observed (Figure 5a), where higher methylation levels were associated with reduced gene expression. Together, these findings suggest that DNA hypermethylation in the upstream regions of the TSS plays a role in the downregulation of gene expression during disuse muscle atrophy. On the other hand, DNA methylation in the downstream region decreased on day 3 post‐HLS and remained low through day 7 (Figure 3a). Studies have reported a negative correlation between downstream hypomethylation and upstream hypermethylation, suggesting that downstream hypomethylation may reduce promoter activity and indirectly facilitate upstream hypermethylation, ultimately contributing to gene expression (Anastasiadi et al., 2018; Kopparapu et al., 2017; Maunakea et al., 2010). These findings demonstrate that downstream DNA hypomethylation occurs during muscle disuse and may be involved in upstream hypermethylation, collectively influencing the downregulation of gene expression.

Reinforcing the observed negative correlation between DNA methylation and gene expression (Figure 5a), FOXO‐dependent upregulated genes during disuse atrophy exhibited hypomethylation in the upstream regions of the TSS on day 7 post‐HLS (Figure 6c,d). On day 3, however, the gene expression of these genes had already increased, despite no significant changes in DNA methylation at this time point (Figure 6c,d). Okamoto et al. reported that FOXO, dephosphorylated in unloaded mouse muscles on day 3 of hindlimb immobilization, is associated with increased expression of atrophy‐related genes such as *Trim63* in both slow‐twitch and fast‐twitch muscle fibers (Okamoto et al., 2011). Considering all these observations, the increased expression of FOXO‐dependent genes during the early stages of disuse likely depends on mechanisms independent of DNA methylation changes and primarily based on the activation of FOXO transcription factors. Hypomethylation in the upstream regions on day 7 post‐HLS thus may function as a secondary mechanism that supports the maintenance of elevated gene expression initiated earlier by FOXO. In contrast, downregulated genes showed no changes in expression on day 3, aligning with the absence of significant methylation changes in this early stage (Figures 2d and 3b). Compared to FOXO‐associated genes, downregulated genes during disuse atrophy appear to initiate their downregulation more heavily through changes in DNA methylation levels in the upstream regions, rather than relying on transcription factors. Although the relationship between transcriptional factors and DNA methylation remains unclear, these findings suggest their interplay in regulating gene expression during muscle atrophy.

Histone marks exhibited dynamic changes during disuse muscle atrophy. On day 3 post‐HLS, the repressive mark H3K27me3 increased in the upstream regions of the TSS (Figure 4a). H3K27me3 contributes to transcriptional repression, and studies in cancer models have demonstrated that H3K27me3 enrichment can precede DNA hypermethylation (Ohm et al., 2007; Schlesinger et al., 2007). Yamagishi et al. reported that chromatin condensation induced by H3K27me3 near the TSS facilitates the recruitment of DNA methyltransferases, leading to CpG site methylation that persists even after H3K27me3 levels decline, thereby maintaining long‐term gene repression (Yamagishi et al., 2024). Consistent with these findings, our study observed a decrease in H3K27me3 levels on day 7 of HLS, coinciding with an increase in DNA methylation (Figures 3a and 4a). These observations suggest that the early accumulation of H3K27me3 in the upstream regions may act to prime these regions for subsequent DNA hypermethylation during disuse muscle atrophy. H3K4 methylation is closely linked to gene expression responses to exercise in the skeletal muscle of humans and mice (Lim et al., 2020; Shimizu & Kawano, 2022). Li et al. recently demonstrated that this modification, through an exercise‐induced increase in the H3K4me3/H3K27me3 ratio around the TSS, facilitates the upregulation of gene expression (Li et al., 2024). Although a similar H3K4me3 increase was observed on day 7 post‐HLS, it showed no correlation with gene expression levels, which instead decreased at that time (Figures 2d, 4a, and 5a). These findings suggest that the disuse‐induced increase in the H3K4me3/H3K27me3 ratio does not directly regulate gene expression during muscle atrophy. Given that H3K4me3 is more commonly associated with transcriptional elongation than initiation (Wang et al., 2023), the observed downregulation may be involved in mechanisms independent of transcriptional elongation. However, the role of H3K4me3 in regulating gene expression during disuse muscle atrophy remains unclear and warrants further investigation.

The present study had certain limitations in scope, as it investigated epigenetic shifts associated with gene expression downregulation prior to and during disuse muscle atrophy using a group‐level analytical approach focused on targeted gene sets. For instance, we did not analyze the gastrocnemius muscle, which undergoes earlier atrophy and differs in fiber type composition from the vastus intermedius (Bloemberg & Quadrilatero, 2012; Hitomi et al., 2005); such comparisons would yield important insights into fiber type‐specific epigenetic responses. Likewise, although our primary focus was on downregulated genes, further analysis of the significantly upregulated genes in Figure 2a,b would provide insight into divergent temporal expression trajectories among genes during disuse‐induced muscle atrophy (Stevenson et al., 2003). Incorporating gene‐ or region‐specific analyses, such as CpG island targeting, would also refine the understanding of how DNA methylation relates to transcriptional control. While not the primary focus of the present study, these directions offer valuable opportunities for future research to build upon the shared regulatory patterns identified here.

## 5 CONCLUSION

The present study investigated the relationship between epigenetic alterations and the downregulation of gene expression during disuse muscle atrophy, focusing on their sequential links. At the onset of atrophy, downregulation of homeostatic gene expression was linked to DNA hypermethylation in the upstream regions of the TSS. Notably, H3K27me3 enrichment was observed in these regions during the pre‐atrophic stage, a phase marked by the absence of both hypermethylation and gene downregulation. Furthermore, FOXO‐regulated upregulated gene expression at the onset of atrophy coincided with DNA hypomethylation in the upstream regions, underscoring the consistent relationship between DNA methylation levels and gene expression. These findings suggest that DNA hypermethylation, which is preceded by H3K27me3 enrichment, is linked to the downregulation of gene expression during disuse muscle atrophy. However, as this study is based on correlative analyses, further investigations using functional approaches are required to determine the causal mechanisms underlying these epigenetic modifications in muscle atrophy. Future studies incorporating other skeletal muscles, finer temporal resolution, and gene‐specific analyses would also help clarify the generality and mechanistic basis of these epigenetic changes.

## FUNDING INFORMATION

Japan Society for the Promotion of Science Fellows, Grant Number: 23KJ2061 (to JS).

## CONFLICT OF INTEREST STATEMENT

The authors declare that they have no competing interests.

## Data Availability

The data that support the findings of this study are available from the corresponding author upon reasonable request.
